# Impact of Vibration on Skin Blood Flow: A Scoping Review

**DOI:** 10.7759/cureus.113301

**Published:** 2026-07-24

**Authors:** Rayya Harb, Rahi Patel, Julianna Foss, William Zagrodzky, Ian Raynshteyn, Harvey N Mayrovitz

**Affiliations:** 1 Osteopathic Medical School, Nova Southeastern University Dr. Kiran C. Patel College of Osteopathic Medicine, Davie, USA; 2 Medical Education, Nova Southeastern University Dr. Kiran C. Patel College of Allopathic Medicine, Davie, USA

**Keywords:** finger blood flow, microcirculation, occupational vibration exposure, peripheral circulation, skin blood flow, vascular response, vibration therapy, whole-body vibration, wound healing

## Abstract

Vibration forces have been reported to alter skin blood flow (SBF) and have been proposed as a noninvasive therapy for modulating exercise performance, wound healing, and thermoregulation. Although numerous studies have explored the effects of vibration on SBF, the direct effects of vibration therapy (VT) on SBF remain inconsistently characterized. This scoping review maps the existing evidence on the effects of localized and whole-body vibration (WBV) on SBF across clinical, laboratory, occupational, and therapeutic settings. The review was conducted using the Arksey and O’Malley Framework, with an initial search for “vibration” AND “skin” AND “blood” across three databases: Embase, Ovid Medline, and Web of Science. Studies included were peer-reviewed, English-language human studies published through October 2025 that evaluated the effects of vibration on SBF. Following a systematic screening process, 46 papers met the inclusion criteria. Of those, 26 (56.5%) demonstrated an overall increase in SBF with exposure to WBV or lower-extremity vibration. Fifteen studies reported decreases in SBF/FBF when examining finger blood flow after hand-transmitted or occupational vibration exposure. Five additional studies reported no significant change, mixed findings, or context-dependent responses, including alterations in reactive hyperemia or indirect measures of perfusion. Changes in SBF were influenced by several factors, including the anatomical location of vibration application, vibration duration, vibration frequency, underlying pathology, and exposure duration. Based on the findings, it is concluded that vibration alters SBF in a context-dependent manner, influenced by anatomic location, vibration frequency, amplitude, and duration of exposure, with hand vibration typically reducing SBF and lower extremity application increasing SBF. Standardized protocols are needed to further elucidate the mechanism by which vibration affects and modulates SBF.

## Introduction and background

Studies suggest that various forms of mechanical stress applied to the skin can enhance blood flow, leading to physiological benefits. For example, scalp massage has been reported to increase local blood flow and may improve hair growth [1]. Vibration is another mechanical force that has gained increasing interest as a noninvasive therapy with applications in neurological rehabilitation, exercise performance, and tissue function [2]. Studies have linked vibration to wound healing, an effect thought to be mediated through improvements in skin blood flow (SBF) [3]. SBF is also relevant to thermoregulation [4], aging skin [5], and autoimmune conditions such as psoriasis [6]. These associations provide clinical motivation for understanding the effects of vibration on SBF, although they do not constitute direct evidence regarding the microcirculatory responses to vibration.

The term vibration therapy (VT) describes the application of mechanical oscillations to the body at a specific frequency and amplitude, either globally through whole-body vibration (WBV) or locally, targeting specific muscles or joints [7]. VT has been described as oscillating or linear in delivery, with linear vibration reported to improve jump height [8,9]. However, not all vibration exposure is therapeutic in intent. Vibration associated with occupational exposure (e.g., a jackhammer) represents a distinct paradigm. Chronic occupational exposure has been linked to adverse vascular outcomes, including reductions in digital blood flow and vibration white finger, contributing to regulatory limits on exposure duration in workplace settings enforced by the Occupational Safety and Health Administration (OSHA). These opposing vascular effects underscore the importance of distinguishing between therapeutic and occupational vibration when interpreting literature, as they differ fundamentally in their intent, exposure characteristics, and expected physiological consequences.

Vibration exerts opposing effects on cutaneous perfusion through two competing mechanisms whose dominance depends on the anatomical site of application. In the lower extremities, mechanical oscillations generate shear stress on vascular endothelial cells, activating endothelial nitric oxide synthase (eNOS) and promoting nitric oxide (NO)-mediated vasodilation [10,11]. Concurrently, myogenic control mechanisms, which reflect the intrinsic tendency of vascular smooth muscle to contract or relax in response to changes in transmural pressure, are engaged and contribute to local vasodilation and increased SBF [12]. In contrast, when vibration is applied to the hands and fingers, it preferentially activates densely distributed mechanoreceptors, such as Pacinian corpuscles, whose engagement is directly correlated with reductions in digital blood flow and is mediated by a centrally driven sympathetic reflex, producing bilateral vasoconstriction in both the exposed and contralateral hands [13]. At the molecular level, acute vibration upregulates α₂C-adrenergic receptor-mediated constriction in cutaneous arteries, a mechanism implicated in vibration-induced vascular disease [14]. Because these pathways produce opposing hemodynamic effects, the anatomical site of vibration application is a critical determinant of the vascular response, a distinction that organizes the findings presented in this review.

Despite increasing interest in vibration-based interventions, the existing literature is characterized by substantial methodological heterogeneity, including variability in vibration frequency, amplitude, duration, anatomical application site, participant population, and methods used to quantify SBF. This heterogeneity limits the ability to draw firm conclusions and precludes meaningful quantitative pooling of results. It is also unclear where the boundary lies between adverse occupational exposure and therapeutic benefit, and no scoping review on this topic has been conducted. These gaps highlight the need for a scoping review to map and characterize the current evidence on the effects of vibration of SBF, including its proposed mechanism of action, modalities, intensities, potential therapeutic effects, and contraindications. The substantial heterogeneity across studies in vibration frequency, amplitude, duration, anatomic site, population, and measurement method makes a comprehensive evidence map, rather than quantitative synthesis, the most appropriate next step.

## Review

Study design and search strategy

This scoping review was conducted using the Arksey and O’Malley Framework [15] and reported in accordance with the Preferred Reporting Items for Systematic Reviewers and Meta-Analyses extension for Scoping Reviews (PRISMA-ScR) [16]. It was conducted using an initial search string “vibration” AND “skin” AND “blood”. This string was applied across three databases (Embase, Ovid Medline, and Web of Science) to identify relevant articles addressing the review question. Based on the titles, abstracts, and keywords, additional search terms were implemented. These terms were incorporated into the final search strategy, the details of which are documented in the supplementary appendix exactly as executed and further refined using each database’s term finder to identify the controlled vocabulary (e.g., Medical Subject Headings (MeSH), Emtree). The final search strategy used Boolean terms and truncation symbols. The search strategy was developed using title and abstract text words, keywords, and index terms, following the Population, Concept, Context (PCC) framework. To cover a wide range of relevant research, a broad set of keywords was used. Vibration therapy-related terms included “electrovibratory,” “oscillation,” “percussion,” and “massage gun therapy.” These terms were combined with skin blood flow terms such as “microcirculation,” “vascularization,” “vessel,” “capillary,” “blood flow distribution,” “blood stream.” Boolean operators (AND, OR) and truncation (*) were incorporated. The core search string applied across databases was: (vibration OR vibration therapy) AND (blood OR skin blood flow) AND (skin OR dermis OR epidermis). The search strings are indicated in Table 1.

**Table 1:** Summary of Search Strings This table presents the results of the structured search strategy used to identify relevant studies examining the effects of vibration on skin blood flow. The search strategy was developed using title and abstract text words, keywords, and index terms, following the Population, Concept, Context (PCC) framework. Search terms were derived from the initial search string “vibration” AND “skin” AND “blood” and conducted across Embase, Ovid Medline, and Web of Science, including Boolean operators and data-base specific subject headings. The number of records retrieved from each search in each data base is presented. This search was conducted using the Arksey and O’Malley framework [15].

Set #	Search Strategy	Results
EMBASE 10/9/25		
1	'vibration'/exp OR 'vibration therapy'/exp	115713
2	'electrovibrat* massage*':ab,ti,kw OR 'hand-arm vibrat*':ab,ti,kw OR 'hand arm vibrat*':ab,ti,kw OR 'high frequency oscillat*':ab,ti,kw OR 'local vibration*':ab,ti,kw OR 'low-intensity vibration*':ab,ti,kw OR 'massage gun application*':ab,ti,kw OR 'massage gun percussion*':ab,ti,kw OR 'massage gun therap*':ab,ti,kw OR 'microvibrat* therap*':ab,ti,kw OR 'oscillat* potential':ab,ti,kw OR 'oscillat* flow':ab,ti,kw OR 'oscillat* motion':ab,ti,kw OR 'oscillat* movement':ab,ti,kw OR 'oscillat* process*':ab,ti,kw OR 'percuss* massage*':ab,ti,kw OR 'percuss* therap*':ab,ti,kw OR 'percuss* treatment*':ab,ti,kw OR 'percuss* massage therap*':ab,ti,kw OR 'percuss* massage treatment*':ab,ti,kw OR 'percussive massage*':ab,ti,kw OR 'percussive massage therapy':ab,ti,kw OR 'percussive massage treatment':ab,ti,kw OR 'vibration':ab,ti,kw OR 'vibration therapy':ab,ti,kw OR 'vibrat*':ab,ti,kw OR 'vibrat* massage*':ab,ti,kw OR 'vibrat* therap*':ab,ti,kw OR 'vibrat therapy*':ab,ti,kw OR 'vibrating therap*':ab,ti,kw OR 'vibration response*':ab,ti,kw OR 'vibro-massage*':ab,ti,kw OR 'vibroacoustic massage*':ab,ti,kw OR 'vibroacustic massage*':ab,ti,kw OR 'vibromassage*':ab,ti,kw OR 'vibrotherap*':ab,ti,kw OR 'whole body vibrat*':ab,ti,kw OR 'whole body vibrat* exercise*':ab,ti,kw OR 'whole body vibrat* therap*':ab,ti,kw OR 'whole body vibrat* training*':ab,ti,kw OR 'wholebody vibrat*':ab,ti,kw OR 'wholebody vibrat* therap*':ab,ti,kw OR 'wholebody vibrat* exercise*':ab,ti,kw OR 'wholebody vibrat* training*':ab,ti,kw OR 'wholebody vibration*':ab,ti,kw OR 'wbv exercise*':ab,ti,kw OR 'wbv therap*':ab,ti,kw OR 'wbv training*':ab,ti,kw OR 'wbve':ab,ti,kw OR 'wbvt':ab,ti,kw	107765
3	'blood'/exp OR 'skin blood flow'/exp	3085253
4	'blood*':ab,ti,kw OR 'human blood*':ab,ti,kw OR 'human peripheral blood*':ab,ti,kw OR 'peripheral blood*':ab,ti,kw OR 'tissue blood*':ab,ti,kw OR 'sanguis':ab,ti,kw OR 'capillar* blood*':ab,ti,kw OR 'arter* blood*':ab,ti,kw OR 'ven* blood*':ab,ti,kw OR 'blood cell*':ab,ti,kw OR 'blood flow distribution*':ab,ti,kw OR 'blood flow estimation*':ab,ti,kw OR 'blood stream*':ab,ti,kw OR 'blood vessel flow*':ab,ti,kw OR 'bloodflow*':ab,ti,kw OR 'regional blood flow*':ab,ti,kw OR 'vascul* flow*':ab,ti,kw OR 'blood flow*':ab,ti,kw OR 'arter* blood flow*':ab,ti,kw OR 'arter* flow*':ab,ti,kw OR 'capillar* flow*':ab,ti,kw OR 'capillar* circulation*':ab,ti,kw OR 'ven* flow*':ab,ti,kw OR 'ven* outflow*':ab,ti,kw OR 'tissue blood flow*':ab,ti,kw OR 'blood circulation*':ab,ti,kw OR 'microcirculat*':ab,ti,kw OR 'skin blood flow*':ab,ti,kw OR 'skin circulation*':ab,ti,kw OR 'cutaneous blood flow*':ab,ti,kw OR 'cutaneous circulation*':ab,ti,kw OR 'dermal blood flow*':ab,ti,kw OR 'dermal circulation*':ab,ti,kw OR 'skin blood circulation*':ab,ti,kw OR 'skin blood supply':ab,ti,kw OR 'skin vascularisation*':ab,ti,kw OR 'skin vascularization*':ab,ti,kw OR 'subcutaneous blood flow*':ab,ti,kw OR 'skin blood vessel*':ab,ti,kw OR 'skin capillar*':ab,ti,kw OR 'skin arter*':ab,ti,kw OR 'skin vasculature':ab,ti,kw OR 'skin vessel*':ab,ti,kw OR 'cutaneous blood vessel*':ab,ti,kw OR 'cutaneous capillar*':ab,ti,kw OR 'dermal capillar*':ab,ti,kw OR 'integumentary blood flow*':ab,ti,kw	3667388
5	'skin'/exp	498175
6	'cutis':ab,ti,kw OR 'derma':ab,ti,kw OR 'human skin':ab,ti,kw OR 'skin layer*':ab,ti,kw OR 'skin':ab,ti,kw OR 'dermis':ab,ti,kw OR 'epidermis':ab,ti,kw OR 'scalp':ab,ti,kw OR 'skin epithelium':ab,ti,kw OR 'skin structure*':ab,ti,kw OR 'cutaneous':ab,ti,kw OR 'keratinocyte*':ab,ti,kw OR 'dermoepidermal junction':ab,ti,kw OR 'papillary dermis':ab,ti,kw OR 'reticular dermis':ab,ti,kw OR 'cuticle':ab,ti,kw OR 'skin surface':ab,ti,kw OR 'epidermal cell*':ab,ti,kw OR 'epidermis cell*':ab,ti,kw OR 'corium':ab,ti,kw OR 'stratum basale':ab,ti,kw OR 'stratum corneum':ab,ti,kw OR 'stratum granulosum':ab,ti,kw OR 'stratum lucidum':ab,ti,kw OR 'stratum spinosum':ab,ti,kw OR 'cutaneous cell*':ab,ti,kw OR 'dermal cell*':ab,ti,kw OR 'dermis cell*':ab,ti,kw OR 'skin cell*':ab,ti,kw OR 'glabrous skin':ab,ti,kw OR 'integumentary system':ab,ti,kw OR 'hair follicle':ab,ti,kw	1301437
7	#1 OR #2	175471
8	#3 OR #4	5765702
9	#5 OR #6	1432101
10	#7 AND #8 AND #9	983
Ovid Medline 10/9/25		
1	exp Vibration/	29106
2	("electrovibrat* massage*" or "electrovibratory massage" or "hand-arm vibrat*" or "hand arm vibrat*" or "high frequency oscillat*" or "high frequency oscillation" or "local vibration*" or "low-intensity vibration*" or "massage gun application*" or "massage gun percussion*" or "massage gun therapy*" or "microvibrat* therap*" or "microvibration therapy" or "oscillatory potential" or "oscillat* flow" or "oscillat* motion" or "oscillat* movement" or "oscillat* process" or "percuss* massage*" or "percuss* therap*" or "percuss* treatment*" or "percuss* massage therap*" or "percuss* massage treatment*" or "percussive massage" or "percussive massage therapy" or "percussive massage treatment" or "percussive therapy" or "percussive treatment" or "Vibration" or "Vibration therapy" or "vibrat*" or "vibrat* massage*" or "vibrat* therap*" or "vibrat massage*" or "vibrat therapy*" or "vibrating massage*" or "vibrating therap*" or "vibration massage*" or "vibration response" or "vibration therap*" or "vibrational massage*" or "vibrational therap*" or "vibraty massage*" or "vibraty therap*" or "vibro-massage*" or "vibroacoustic massage*" or "vibroacustic massage*" or "vibromassage*" or "vibrotherap*" or "whole body vibrat*" or "whole body vibrat* exercise*" or "whole body vibrat* therap*" or "whole body vibrat* training*" or "whole body vibration*" or "whole body vibration training" or "wholebody vibrat*" or "wholebody vibrat* therap*" or "wholebody vibrat* exercise*" or "wholebody vibrat* training*" or "wholebody vibration*" or "WBV exercise*" or "WBV therap*" or "WBV training*" or "WBVE" or "WBVT").ab,ti,kf.	113080
3	Exp Blood/	53897
4	("Blood*" or "Human blood*" or "Human peripheral blood*" or "Peripheral blood*" or "Tissue blood*" or "Sanguis" or "Capillar* blood*" or "Arter* blood*" or "Ven* blood*" or "Blood cell*" or "Blood flow distribution*" or "Blood flow estimation*" or "Blood stream*" or "Blood vessel flow*" or "Bloodflow*" or "Regional blood flow*" or "Vascul* flow*" or "Blood flow*" or "Arter* blood flow*" or "Arter* flow*" or "Capillar* flow*" or "Capillar* circulation*" or "Ven* flow*" or "Ven* outflow*" or "Tissue blood flow*" or "Blood circulation*" or "Microcirculat*" or "skin blood flow*" or "skin circulation*" or "cutaneous blood flow*" or "cutaneous circulation*" or "dermal blood flow*" or "dermal circulation*" or "skin blood circulation*" or "skin blood supply" or "skin vascularisation*" or "skin vascularization*" or "subcutaneous blood flow*" or "skin blood vessel*" or "skin capillar*" or "skin arter*" or "skin vasculature" or "skin vessel*" or "cutaneous blood vessel*" or "cutaneous capillar*" or "dermal capillar*" or "integumentary blood flow*").ab,ti,kf.	2520461
5	exp Skin/ or exp Dermis/ or exp Epidermis/	259334
6	("cutis" or "derma" or "human skin" or "skin layer*" or "skin" or "dermis" or "epidermis" or "scalp" or "skin epithelium" or "skin structure*" or "cutaneous" or "keratinocyte*" or "dermoepidermal junction" or "papillary dermis" or "reticular dermis" or "cuticle" or "skin surface" or "epidermal cell*" or "epidermis cell*" or "corium" or "stratum basale" or "stratum corneum" or "stratum granulosum" or "stratum lucidum" or "stratum spinosum" or "cutaneous cell*" or "dermal cell*" or "dermis cell*" or "skin cell*" or "glabrous skin" or "integumentary system" or "hair follicle").ab,ti,kf.	934654
7	1 or 2	120410
8	3 or 4	2538286
9	5 or 6	1004248
10	7 and 8 and 9	312
Web of Science 10/9/25		
1	TS=("electrovibrat* massage*" OR "electrovibratory massage" OR "hand-arm vibrat*" OR "hand arm vibrat*" OR "high frequency oscillat*" OR "high frequency oscillation" OR "local vibration*" OR "low-intensity vibration*" OR "massage gun application*" OR "massage gun percussion*" OR "massage gun therapy*" OR "microvibrat* therap*" OR "microvibration therapy" OR "oscillatory potential" OR "oscillat* flow" OR "oscillat* motion" OR "oscillat* movement" OR "oscillat* process" OR "percuss* massage*" OR "percuss* therap*" OR "percuss* treatment*" OR "percuss* massage therap*" OR "percuss* massage treatment*" OR "percussive massage" OR "percussive massage therapy" OR "percussive massage treatment" OR "percussive therapy" OR "percussive treatment" OR "Vibration" OR "Vibration therapy" OR "vibrat*" OR "vibrat* massage*" OR "vibrat* therap*" OR "vibrat massage*" OR "vibrat therapy*" OR "vibrating massage*" OR "vibrating therap*" OR "vibration massage*" OR "vibration response" OR "vibration therap*" OR "vibrational massage*" OR "vibrational therap*" OR "vibraty massage*" OR "vibraty therap*" OR "vibro-massage*" OR "vibroacoustic massage*" OR "vibroacustic massage*" OR "vibromassage*" OR "vibrotherap*" OR "whole body vibrat*" OR "whole body vibrat* exercise*" OR "whole body vibrat* therap*" OR "whole body vibrat* training*" OR "whole body vibration*" OR "whole body vibration training" OR "wholebody vibrat*" OR "wholebody vibrat* therap*" OR "wholebody vibrat* exercise*" OR "wholebody vibrat* training*" OR "wholebody vibration*" OR "WBV exercise*" OR "WBV therap*" OR "WBV training*" OR "WBVE" OR "WBVT")	682132
2	TS=("Blood*" OR "Human blood*" OR "Human peripheral blood*" OR "Peripheral blood*" OR "Tissue blood*" OR "Sanguis" OR "Capillar* blood*" OR "Arter* blood*" OR "Ven* blood*" OR "Blood cell*" OR "Blood flow distribution*" OR "Blood flow estimation*" OR "Blood stream*" OR "Blood vessel flow*" OR "Bloodflow*" OR "Regional blood flow*" OR "Vascul* flow*" OR "Blood flow*" OR "Arter* blood flow*" OR "Arter* flow*" OR "Capillar* flow*" OR "Capillar* circulation*" OR "Ven* flow*" OR "Ven* outflow*" OR "Tissue blood flow*" OR "Blood circulation*" OR "Microcirculat*" OR "skin blood flow*" OR "skin circulation*" OR "cutaneous blood flow*" OR "cutaneous circulation*" OR "dermal blood flow*" OR "dermal circulation*" OR "skin blood circulation*" OR "skin blood supply" OR "skin vascularisation*" OR "skin vascularization*" OR "subcutaneous blood flow*" OR "skin blood vessel*" OR "skin capillar*" OR "skin arter*" OR "skin vasculature" OR "skin vessel*" OR "cutaneous blood vessel*" OR "cutaneous capillar*" OR "dermal capillar*" OR "integumentary blood flow*")	2720891
3	TS=("cutis" OR "derma" OR "human skin" OR "skin layer*" OR "skin" OR "dermis" OR "epidermis" OR "scalp" OR "skin epithelium" OR "skin structure*" OR "cutaneous" OR "keratinocyte*" OR "dermoepidermal junction" OR "papillary dermis" OR "reticular dermis" OR "cuticle" OR "skin surface" OR "epidermal cell*" OR "epidermis cell*" OR "corium" OR "stratum basale" OR "stratum corneum" OR "stratum granulosum" OR "stratum lucidum" OR "stratum spinosum" OR "cutaneous cell*" OR "dermal cell*" OR "dermis cell*" OR "skin cell*" OR "glabrous skin" OR "integumentary system" OR "hair follicle")	1127842
4	#1 AND #2 AND #3	467

Eligibility criteria

This review is limited to studies of humans who experienced vibration, in which skin blood flow was measured before and after its application. The articles needed to be published in a peer-reviewed English-language journal, with publication dates up to October 2025. There were no cultural or geographic limits on the inclusion criteria. Excluded were opinion pieces, editorials, review articles, and conference papers or abstracts.

Study selection process

**Figure 1:**
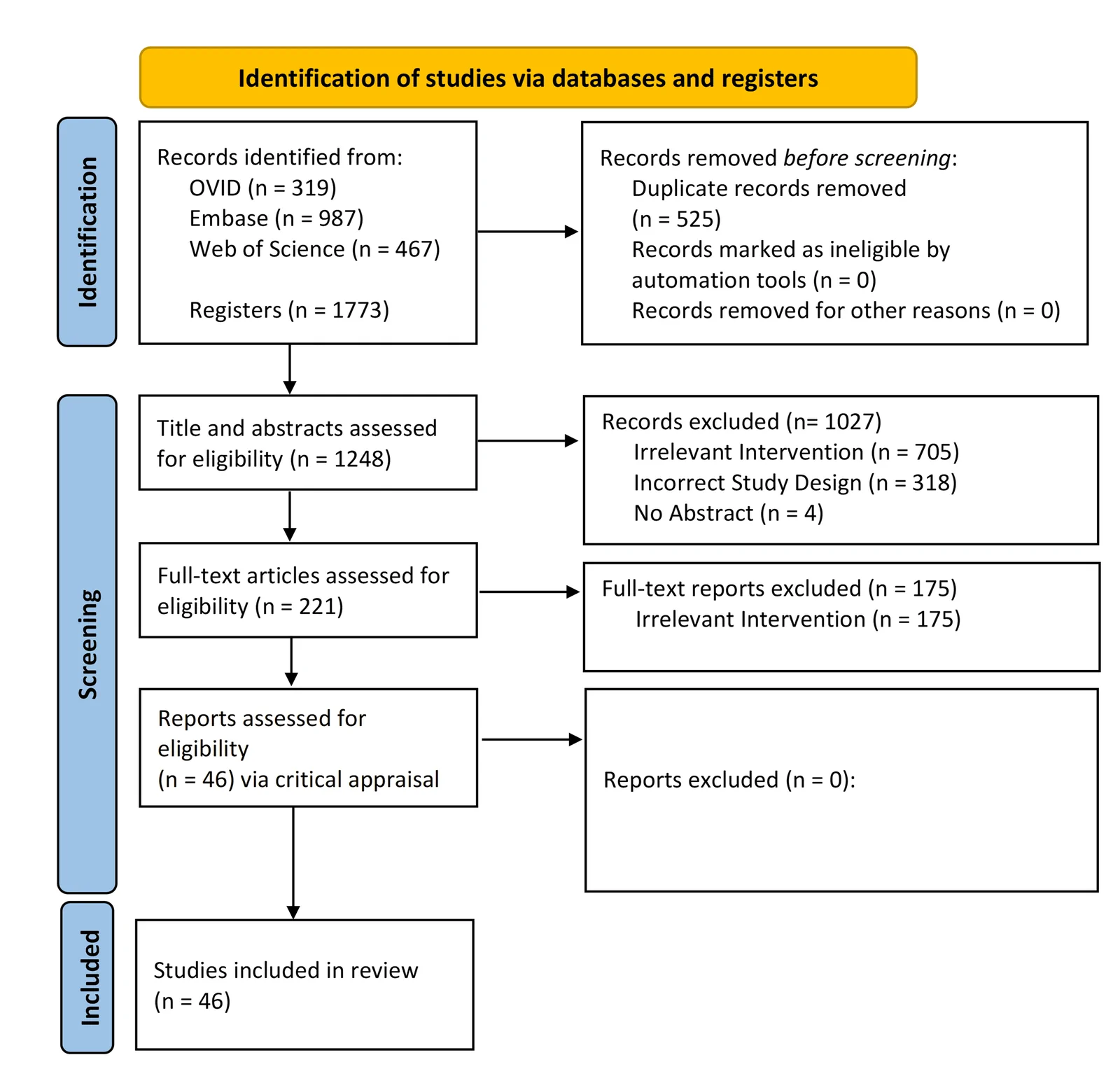
PRISMA search strategy PRISMA-ScR: Preferred Reporting Items for Systematic reviews and Meta-Analyses extension for Scoping Reviews

The initial search conducted on September 10, 2025, retrieved a total of 1773 articles from the three selected databases. Utilizing the Rayyan platform (Rayyan Systems, Inc., Cambridge, MA, USA), duplicates were removed, and abstracts were prepared for screening. After removing 525 duplicates, 1248 articles were screened for eligibility by two independent reviewers by looking at the title and abstract using the criteria described above. This resulted in 1026 articles being excluded due to irrelevant interventions, incorrect study design, and/or lack of an abstract. The resulting 221 articles were assessed in full by the same two independent reviewers for relevance to the study design according to the inclusion and exclusion criteria. After the full-text screening, 175 articles were excluded. The remaining 46 articles met all criteria and were appraised for use in the review. The most common exclusion criterion was an intervention deemed irrelevant. Citations were managed throughout the review process by EndNote. The complete selection process is illustrated below in the PRISMA-ScR flowchart in Figure 1 [16]. The protocol for this review was not prospectively registered, which is consistent with the Arksey and O’Malley scoping review framework [15]. 

Data quality appraisal

In this review, each article was systematically appraised for experimental validity, patient profiles, potential confounding variables, and clarity of intervention using the Joanna Briggs Institute (JBI) Critical Appraisal Tools [17]. Papers were sequentially reviewed using the appropriate appraisal tool for each study design. Each article was independently assessed by two reviewers, and their decisions regarding the strength of each article were compared. Disagreements between reviewers, though infrequent, were resolved through collaborative discussion in all cases, with consensus reached prior to inclusion in the final appraisal.

Data extraction was performed independently by two reviewers for all 46 included studies. The extracted data included study design, participant characteristics, presence of underlying health conditions, vibration modality, frequency, amplitude, exposure duration, anatomical application site, SBF measurement method and location, and primary outcome. Although a formally standardized extraction form was not used, both reviewers extracted data using a consistent set of predetermined variables corresponding to different categories. Discrepancies between reviewers were resolved through a discussion with a comparison of extracted data, with consensus reached on all items prior to inclusion in the final table.

Studies were categorized as demonstrating increased, decreased, or mixed/no significant change in SBF based on the statistical significance of outcomes as reported by the original study authors (typically defined as p<0.05); studies without formal significance testing were categorized based on the authors' own stated interpretation of their findings. Effect size magnitude was not used as a categorization criterion; studies were classified based on the direction and statistical significance of the reported outcome only.

Given substantial heterogeneity in vibration modality, frequency, amplitude, exposure duration, anatomical site, and outcome measurement across the included studies, a narrative synthesis was employed rather than quantitative meta-analysis, consistent with recommended approaches for scoping reviews addressing heterogeneous evidence bases.

Results

Overall, this review includes 46 studies that encompass a diverse patient population and a variety of experimental approaches. Of these, 23 (50%) reported a vibration-related increase in skin blood flow, 20 (43.5%) reported decreases in skin or finger blood flow, and three (6.5%) demonstrated no significant change or context-dependent responses. Among the 23 papers reporting a vibration-related increase in skin blood flow, 12 studies (52.2%) used whole-body vibration (WBV) with healthy participants, four (17.4%) included participants with underlying conditions, and seven (30.4%) reported increases in skin blood flow using various local vibration techniques.

Table 2 summarizes the key aspects of each study and its outcome, including study design, number of participants, whether a health condition was present in the population studied, SBF measurement method and location, vibration site and frequency, study goal, and main outcome.

**Table 2:** Summary of the Included Articles Column 1 indicates the first author, publication date, and reference number. The subjects (Subj) are classified as either healthy normal (H) or having some medical condition (M). The other abbreviations used in the table are as follows. Experimental (Exp); Randomized control trial (RCT); Cross-sectional experimental (CSE); Experimental crossover (EC); Crossover Design (XO); Quasi-Experimental Design (QED); Matched Case-Control + Repeated Measures (MCC-RM), Observational (OBS); Experimental Observational (Exp OBS); Double Blind (BD); Repeat Measure (RM); Laboratory Controlled (LC), Single-Blind (SB); Low-intensity vibration (LIV); Whole body vibration exercise (WBVE); Whole-body vibration (WBV); Local Vibration (LV); Dynamic infrared thermography (DIRT); Skin temperature probe (STP); Infrared Thermography (IRT); Skin temperature (ST); Laser Speckle Flowgraphy System (LSFG); Transcutaneous oxygen tension (TcPO2); Laser Doppler Imager (LDI), Near-infrared spectroscopy (NIRS); Muscle Oxygenation (SmO2); Photoelectric Plethysmography (PPG), Plethysmography Strain-Gauge (SGPG); Finger blood flow (FBF); Lower Extremity (LE); Laser Doppler Flowmetry (LDF), Thermal Diffusion Flowmetry (TDF), Blood Volume (BV). F=female; M=male; y=years; BMI=body mass index; CVD=cardiovascular disease; RLS/WED=restless legs syndrome/Willis-Ekbom disease.

Author Year [##]	Study design	N	Study population	Medical condition present	SBF measurement method and location	Vibration site and frequency	Study goal	Main outcome
Tzen et al. 2018 [18]	Exper	9	5F/4M; 30-55 y; healthy adults	No	LDF - Direct foot dorsum	Feet WBV 30 Hz	To investigate the impact of LIV on SBF in healthy human subjects	The effects of LIV resulted in an acute increase in SBF, which returns to baseline after conclusion of the vibration
Elfering et al. 2013 [19]	Exper	23	23F; 18-30 y; BMI 17-26 kg/m²; non-athletes	No	LDF - Direct LDF trapezius	Feet WBV 6 Hz	To evaluate acute effects of WBV on back musculature and middle back SBF in relation to the prevention of musculoskeletal disorders	In comparison to the baseline and sham condition, SBF increased substantially in the middle back
Mahbub et al. 2020 [20]	Exper	30	15M/15F; ≥65 y; healthy older adults; no diabetes	No	LSFG - Direct dorsal foot	Feet WBV 15 Hz, 20 Hz, 25 Hz	To investigate the acute effects of WBV on peripheral circulation, cutaneous sensation, and balance	Dorsal SBF increased significantly under the 20 Hz and 25 Hz conditions, with a greater effect observed at 25 Hz; a minimum WBV frequency of 20 Hz was required to improve peripheral blood flow
Lohman et al. 2011 [21]	EC	10	5M/5F; 20-30 y; healthy adults	No	LDF - Direct gastrocnemius	Feet WBV 50 Hz	To evaluate the individual and combined effects of vibration and moist heat on SBF and ST	Applying both vibration and moist heat yielded the most substantial increase of SBF and second highest increase in SBF nine minutes following application
Lohman et al. 2007 [22]	RCT	45	22F/23M; 18-43 y; healthy adults; no circulatory disorders	No	LDF - Direct distal lower leg	Feet WBV 30 Hz	To assess the short-term effects of high-intensity isometric weight-bearing exercise and vibration alone on SBF	Short periods of vibration alone cause a significantly increased SBF in individuals with healthy microcirculation for at least 10 mins post-intervention
Lohman et al. 2012 [23]	Exper	10	3F/7M; 55-73 y; healthy older adults; excluded diabetes, CVD, orthopedic conditions	No	LDF - Direct posterior calf	Feet WBV 50 Hz	To compare how short-term vibration affects LE SBF and temperature with those of moist heat and massage heating pads	The combination of moist heat and vibration produced the greatest increase in SBF of all interventions
Maloney-Hinds et al. 2008 [24]	Exper Mixed	25	Study 1: 11M/7F healthy adults; Study 2: 3M/4F healthy adults	No	LDF - Direct underside of forearm	Forearm WBV 30 Hz 50 Hz	To determine the effects of passive forearm vibration at 30 Hz versus 50 Hz on SBF and identify the optimal duration of application	Five minutes of vibration at either 30 or 50 Hz significantly increased SBF, with a faster response observed at 50 Hz; additionally, 50 Hz did not induce vasoconstriction during recovery
Hazell et al. 2008 [25]	EC	8	Healthy recreational men; 20-30 y	No	STP - Indirect (ST) 2.5 cm superior to the left lateral malleolus	Feet WBV 45 Hz	To evaluate the impact of vertical WBV on heart rate, mean arterial pressure, femoral artery blood flow, and leg skin temperature during both rest and static exercise	Seated WBV did not affect leg skin temperature, whereas adding vibration to a static semi-squat resulted in increased leg skin temperature
Games and Sefton 2013 [26]	Exper Repeated measures crossover	14	9F (21.7±2.4 y); 5M (20.8±1.1 y); healthy adults	No	DIRT - Indirect (ST) Lateral to medial edge of gastrocnemius belly	Feet WBV 50 Hz	To investigate immediate WBV impacts on peripheral blood perfusion, muscle oxygenation, motoneuron pool excitability, and sensory nerve conduction velocity	WBV significantly elevated superficial skin temperature and total hemoglobin, while producing no changes in oxyhemoglobin or sural sensory nerve conduction velocity. It also increased deoxyhemoglobin levels and suppressed the soleus Hoffman reflex
Robbins et al. 2014 [27]	Exper Repeated Measures crossover	20	12M/8F; 21-27 y; healthy adults; no recent lower limb disorders	No	STP - Indirect (ST) Tibialis anterior, peroneus longus, extensor hallucis brevis	Feet WBV 40 Hz	To examine cardiovascular responses to WBV during quiet standing and determine whether the central or peripheral cardiovascular system has a greater influence	The findings indicate that the peripheral vascular system is highly sensitive to WBV exposure
Menendez et al. 2015 [28]	Randomized	13	Healthy males; mean age 21.2 y	No	IRT - Indirect (ST) gastrocnemius	Feet WBV 26 Hz	To investigate the acute effects of isolated and combined WBV and electromyostimulation on popliteal arterial blood velocity and calf skin temperature.	The combined use of WBV and ES resulted in greater increases in mean and peak blood velocity compared with either intervention applied independently
Lyons et al. 2022 [29]	RCT	27	15M/12F; healthy adults	No	IRT - Indirect (ST) FLIR camera	Feet WBV 30 Hz, 40 Hz, 45 Hz, 10 Hz, 18 Hz, 26 Hz	To compare blood flow and oxygenation across six treatment parameters using vertical and side-alternating WBV.	Neither vertical nor side-alternating WBV produced meaningful increases in SBF when skin temperature was used as a surrogate measure. No significant time-dependent changes in SBF were observed, and although some frequency-related effects were detected, they were inconsistent and did not demonstrate a clear enhancement in blood flow
Moreira-Marconi et al. 2019 [30]	CSE	22	15F/7M; 20-45 y; healthy adults (physiotherapists, physicians, biologists); excluded musculoskeletal disorders, vertigo, implants, and other clinical diseases	No	IRT - Indirect (ST) Posterior lower limb	Feet WBVE 30 Hz, 40 Hz, 45 Hz, 10 Hz, 18 Hz, 26 Hz	To examine the effects of acute WBVE on skin temperature in lower limb regions.	A 60-second bout of WBVE acutely altered posterior lower limb skin temperature, likely reflecting a reduction in SBF. This response may be due to an increased demand for blood flow to active muscles during WBVE, resulting in blood being redirected away from the skin during the initial phase of exercise
Foster et al. 2020 [31]	Exper, LC	16	7M/9F; 18-38 y; healthy adults; no motion sickness susceptibility	No	PPG - Indirect (BV) Finger	WBV 0.03 Hz, 0.05 Hz, 0.1 Hz, 0.2 Hz	To determine whether slow, low-frequency sinusoidal motion that triggers sopite syndrome affects skin sympathetic nerve activity and blood flow.	The SBF modulation was significantly lower during the low-frequency motion relative to baseline. This shows that sopite syndrome is linked to modifications in sympathetic outflow and vasoconstriction to the skin.
Zhu et al. 2020 [32]	Exper	15	4M/11F; mean age 26.5 y; healthy adults	No	LDF - Direct plantar foot	LV Foot 15 Hz, 20 Hz, 25 Hz	To analyze the influence of LV on plantar SBF response while standing and explore microvascular control mechanisms associated with standing with and without vibration.	LV produced a significant increase in SBF compared with the sham condition and elicited distinct alterations in SBF oscillations across the metabolic, neurogenic, myogenic, respiratory, and cardiac frequency bands
Zhu et al. 2020 [33]	Exper	12	2M/10F; Mean age 25.4 y; healthy adults; no diabetes or CVD	No	LDF - Direct plantar foot	LV Foot 35 Hz 100 Hz	To examine the effects of different vibration frequencies applied to the plantar surface on SBF and the underlying control mechanisms.	Compared with the 35 Hz and 0 Hz conditions, the 100 Hz vibration protocol elicited significantly higher SBF, likely mediated through metabolic endothelial and neurogenic control mechanisms
Liao et al. 2020 [34]	Exper	12	18-35 y; healthy adults; no cardiovascular, neurological, or skin disease	No	LDF - Direct plantar foot	LV Foot 35 Hz, 100 Hz	To determine whether nonlinear dynamics of SBF responses are important for evaluating the effects of LV on SBF	Only the 100 Hz vibration protocol significantly increased multiscale SBF regularity, primarily within the 0.0095-0.15 Hz range, supporting the use of multiscale regularity to assess LV-induced improvements in SBF
Ren et al. 2019 [35]	Exper	26	7M/4F diabetic adults and 6M/9F healthy adults	Yes	LDF - Direct plantar foot	LV plantar foot 50 Hz	To examine the immediate results of local vibration with different treatment durations on plantar SBF responses in diabetic and healthy individuals	Intermittent 50 Hz vibration increased SBF in both diabetic and healthy subjects, while continuous vibration improved SBF only in healthy individuals; diabetic subjects showed weaker SBF responses overall
Clijsen et al. 2025 [36]	OBS	26	Healthy nonsmoking adults >18 y; excluded musculoskeletal injury, diabetes, polyneuropathy, implants, pregnancy, and relevant medications	No	LDF - Direct IRT-Indirect (ST) NIRS -Indirect (SmO2) Anterior thigh	LV Anterior Thigh, 29 Hz	To assess changes over time in local skin temperature, deep tissue perfusion, and muscle oxygenation following a standardized 4-minute Theragun™ treatment of the vastus medialis in healthy women	A 4-minute Theragun™ application improved skin temperature, tissue perfusion, red blood cell movement, and muscle oxygenation in cutaneous, subcutaneous, and muscle tissues
Piotrowska et al. 2022 [37]	QED	57	Healthy females; 22-24 y; grade 1+ cellulite	No	IRT - Indirect (ST) buttock, posterior thigh	LV buttock, posterior thigh 18-39 Hz	To evaluate the effects of vibration therapy on buttock and thigh cutaneous microcirculation and its potential role in reducing cellulite	Skin temperature increased significantly after both the first and final treatments, and cellulite grade significantly decreased in participants with grade 1 and grade 2 cellulite. The 60-minute seated vibration therapy condition produced the greatest effects
Lai et al. 2020 [38]	Exper/XO	23	12M/11F; Healthy novice runners; 20-45 y; ran 1-3×/week	No	LDF - Direct calf	LV Lower limb 20-40 Hz (mixed)	To evaluate the effects of vibrating rollers versus standard foam rollers on local SBF, blood flow oscillation, and muscle recovery.	Vibrating rollers produced a non-significant increase in blood perfusion compared with foam rollers, but generated approximately 30% greater blood flow oscillations associated with endothelial activation
Zhu et al. 2021 [39]	DB, RM, XO	10	10F; 18-45 y; healthy young adults; no diabetes	No	LDF - Direct plantar foot	LV plantar foot 100 Hz	To determine whether preconditioning the plantar foot tissue prior to walking reduces plantar ischemia during ambulation	Compared with the sham condition, vibration resulted in significantly lower peak and total post-intervention SBF, indicating attenuation of the reactive hyperemic response. Reduced SBF following vibration may reflect decreased ischemic burden during walking, supporting local vibration preconditioning as a potential intervention for diabetic foot ulcer prevention
Ren et al. 2021 [40]	Exper, XO	13	5M/8F; mean age 23 y; healthy adults	No	LDF - Direct dorsal foot	LV Foot 50 Hz	To determine whether vibration applied to the foot during occlusive compression reduces the reactive hyperemic response following pressure release	The vibration plus compression condition significantly decreased reactive hyperemia, indicating that vibration may mitigate hyperemic responses in foot tissue under occlusive compression
Ye and Griffin 2011 [41]	Exper XO	12	Healthy males; 25-31 y	No	SGPG - Indirect (BV) Finger	LV Hand 125 Hz	To assess the impact of temperature on vibration-induced changes in finger circulation in healthy individuals and evaluate variability in each response to vibration and temperature	Unilateral hand vibration decreased FBF and skin temperature in the opposite unexposed hand, with effects varying according to temperature
Ye and Griffin 2011 [42]	Exper	40	20M/20F; healthy university students; right-handed	No	SGPG-indirect (BV) finger	LV Hand 125 Hz	To examine whether reductions in FBF are associated with Pacinian channel vibrotactile perception thresholds and how sex may regulate this response	FBF reductions occurred at vibration magnitudes above the perception threshold, with greater magnitudes producing larger decreases in FBF. Women exhibited lower vibrotactile thresholds and greater vibration-induced reductions in FBF, suggesting that mechanoreceptors involved in vibration perception also contribute to vascular responses to vibration
Ye and Griffin 2013 [43]	Exper	15	Healthy males; mean age 24.5 y	No	SGPG - indirect (BV) finger	LV Hand 125 Hz	To determine whether the contact surface area of applied vibration influences reductions in FBF	125 Hz vibration administered exclusively to the right hand resulted in a decrease of the FBF in both hands. Additionally increasing the probe size yielded greater reductions in FBF. Increasing the contact area increases the Pacinian activation and induces greater vasoconstriction.
Luo et al. 2000 [44]	Exper	10	6M/4F; healthy adults	No	LDF - direct finger	LV Hand 60 Hz	To investigate the effects of vibration magnitude and repeated exposure on FBF in healthy individuals under laboratory conditions	Higher vibration levels tended to enhance the decline in FBF in both hands; repeated exposure to vibration had a cumulative contribution to the decrease in FBF in the unexposed left hand
Bovenzi and Griffin 1997 [45]	Exper XO	8	Healthy males; 23-44 y	No	SGPG - Indirect (BV) finger	LV Fingers 31.5 Hz, 125 Hz	To examine changes in digital circulation during and following hand-transmitted vibration at different magnitudes and frequencies, and to determine how hemodynamic responses are influenced by vibration frequency, acceleration, and velocity.	Acute digital circulatory responses to vibration were dependent on both vibration magnitude and frequency, producing complex hemodynamic changes. The observed finger vascular responses did not support the frequency weighting assumptions outlined in ISO 5349
Bovenzi et al. 1995 [46]	XO	8	Healthy males; 23-47 y	No	SGPG - indirect (BV) Finger	LV Hand 125 Hz	To examine the local and systemic pathophysiological responses associated with the acute effects of unilateral vibration on digital circulation in healthy male participants.	Immediately following vibration exposure, there was a brief rise in FBF in the vibrated right finger, whereas vasodilation was absent in the non-vibrated left finger. In both the vibrated and non-vibrated fingers, FBF and FST decreased significantly during the recovery period.
Bovenzi et al. 1998 [47]	Exper RM XO	10	Healthy men, aged 21-45	No	SGPG - indirect (BV) Finger	LV Hand 125 Hz	To evaluate the effects of varying durations of hand-transmitted vibration exposure on finger circulation.	Prolonged hand vibration exposure resulted in larger decreases in FBF in both the vibrated and opposite fingers, involving both local vasoconstriction and sympathetic nervous system responses
Bovenzi et al. 1999 [48]	Exper	10	Healthy males; mean age 32 y	No	SGPG - indirect (BV) finger	LV Hand 125 Hz	To examine the central and local pathophysiological mechanisms underlying the acute effects on the digital circulation in healthy men exposed to unilateral vibration	Localized 125 Hz hand vibration decreased FBF in both exposed and unexposed fingers, with greater vibration magnitudes producing stronger vasoconstrictive responses
Bovenzi et al. 2000 [49]	Exper	10	Healthy males; mean age 31.5 y	No	SGPG - indirect (BV) Finger	LV Hand 16 Hz, 31.5 Hz, 63 Hz, 125 Hz, 250 Hz	To examine alterations in finger circulation during and following acute exposure to progressively increasing magnitudes of hand-transmitted vibration	Hand vibration reduced FBF in both vibration-exposed and unexposed fingers, with vasoconstriction becoming more pronounced at higher vibration frequencies
Bovenzi et al. 2001 [50]	XO	10	Healthy males; student/office workers	No	SGPG-Indirect (BV) Finger	LV Hand 125 Hz	To identify the effects of vibration on finger circulation with different combinations of magnitude and duration that produce the same energy-equivalent acceleration, as per current hand-transmitted vibration standards.	Vibration-induced reductions in FBF were greater in the exposed finger than in the non-exposed finger
Bovenzi et al. 2004 [51]	Exper XO	10	Healthy males; mean age 29 y; no vibrating tool exposure	No	SGPG-Indirect (BV) Finger	LV Hand 125 Hz	To evaluate differences in the immediate effects of continuous and intermittent vibration on finger blood flow with matched total duration and energy equivalent acceleration	Finger-specific vibration causes an immediate vasoconstrictive effect accompanied by decreased blood flow independent of the vibration pattern, although intermittent exposure leads to less pronounced post-vibration blood flow reductions than continuous exposure
Miyakita et al. 1990 [52]	Exper, LC	A: 5 B: 7 C: 12 D: 33	A-C: healthy males, 20-50 y; D: vibration disease patients, 51-79 y	A: No B: No C: No D: Yes	STP - indirect (ST) finger	LV Hand 105 dBa	To assess peripheral circulation during chainsaw operation, with a specific focus on the acute changes in blood caused by the contributions of tool weight and grip force	Exposure to handheld vibration acutely decreases FBF and skin temperature through vasoconstriction, with responses affected by vibration characteristics as well as tool weight, ambient temperature, and grip force
Mahbub and Harada 2008 [53]	Exper	8	Healthy non-smoking normotensive males; mean age 24 y; BMI 21.9	No	TDF - Direct	LV Hand 31.5 Hz, 125 Hz, 250 Hz	To examine the concurrent responses in digital circulation at both palmar and dorsal skin induced by immediate exposure to a vibratory handle, and to evaluate the significance of the measurement site in relation to these responses	Following vibration exposure, blood flow and skin temperature remained significantly elevated in the dorsal finger and reduced in the palmar finger across different frequencies relative to control conditions. Vibration produced significant difference in digital circulation between the palmar and dorsal regions of the finger
Nohara et al. 1986 [54]	XO	5	Healthy males; 25-31 y; no vibration tool exposure	No	Hydrogen gas clearance - direct finger	LV Hand 30 Hz, 60 Hz, 120 Hz, 240 Hz, 480 Hz, 960 Hz	To evaluate the effects of local vibration exposure on peripheral circulatory and nervous functions and determine which system is most impacted	The effects of local vibration on physiological function were frequency dependent. Lower vibration frequencies mainly impacted the peripheral nervous system, whereas the peripheral circulatory system responded to both low and high frequencies. Human outcomes aligned with findings reported in animal models.
Gao and Ye 2022 [55]	Exper XO	12	Healthy males; 22-29 y	No	SGPG - indirect (BV) finger	LV Hand 125 Hz	To analyze the immediate impacts of the hand grip and feed exertions on the vascular system at the fingers while exposed to hand-arm vibration, and determine which type of active force has the most severe effects on vascular function.	Exposure to 125 Hz vibration showed a decrease in FBF, and the extent of vasoconstriction is associated with the subject’s vibration perception threshold.
Maloney-Hinds et al. 2009 [56]	QED	20	10 adults with type 2 diabetes and 10 matched healthy controls (each: 6M/4F; mean age ~56 y); no cardiovascular or neurological disease	Yes	LDF - direct forearm	Forearm LV 50 Hz	Compared the SBF and NO production of healthy adults and adults with type 2 diabetes in response to external vibration of the forearm.	Individuals with diabetes demonstrated lower SBF and reduced nitric oxide responses to externally applied vibration compared with matched healthy controls
Johnson et al. 2014 [57]	EC	12	4M/8F; diabetes and/or peripheral neuropathy	Yes	LDI - direct foot	Feet WBV 26 Hz	To examine the impact of low-frequency, low-amplitude WBV on whole blood nitric oxide concentrations and SBF in patients with distal symmetric polyneuropathy.	Diabetic patients showed improved SBF when using WBV compared to the sham condition
Rodriguez-Reyes et al. 2022 [58]	RCT	50	Adults with type 2 diabetes <6 y duration; 40-70 y; non-smokers; no ulcers or disabling diabetic complications	Yes	TcPO2 - Indirect (O2) feet dorsum	Feet WBV 20 Hz	To determine whether a 12-week WBV intervention improves foot blood flow perfusion.	The experimental group demonstrated a significant increase in TcPO2 compared with the control group, with subjects exposed to WBV and exercise showing an approximate 3 mmHg increase
Alves et al. 2020 [59]	Double-blind pilot RCT	20	3M/17F; 60-80 y; type 2 diabetes	Yes	IRT - Indirect (ST) FLIR camera Feet	Feet WBV 24 Hz	To evaluate the immediate response of a single WBV training session to peripheral ST and peripheral blood flow of older adults with type 2 diabetes	A single session of full-body vibration was associated with decreased peripheral skin temperature and reduced lower-extremity blood flow in older adults with type 2 diabetes.
Mitchell et al. 2016 [60]	Aim 1:XO Aim 2: MCC-RM	12,11	RLS/WED patients and age-/sex-matched healthy controls	Yes/No	LDI - Direct foot dorsum	Feet WBV 26 Hz	To evaluate whether a two-week WBV intervention reduces symptoms associated with RLS/WED and whether symptom improvement is mediated by increased SBF.	Individuals with RLS/WED exhibited lower baseline SBF, but WBV increased blood flow flux to levels comparable to healthy controls. Although a two-week WBV intervention reduced RLS/WED symptoms, these improvements did not appear to be associated with increased resting SBF
Mitchell and Johnson 2014 [61]	Exper	20	10 RLS participants (5M/5F) and 10 age-matched healthy controls (5M/5F)	Yes	LDI - direct foot dorsum	Feet WBV 26 Hz	To investigate whether WBV can relieve individuals with restless leg syndrome (RLS) by improving SBF and if it can induce increases in NO blood concentration	Individuals with RLS demonstrated higher SBF than control subjects and showed a greater increase in blood flow in response to WBV, despite no accompanying rise in nitric oxide concentrations measured from antecubital fossa blood samples
Zheng et al. 2009 [62]	SB, RCT	49	14M/35F; frail older adults; 62-93 y; multiple comorbidities	Yes	Thermistor - Indirect (ST) gastrocnemius	Sinusoidal sound waves WBV 27-113 Hz	To assess the effects of a low-frequency sound wave therapy program on functional capacity, blood circulation, and bone metabolism in frail older adults	Low-frequency sound wave therapy may enhance functional capacity and well-being in frail older adults, with vibration increasing SBF and transiently lowering blood pressure during application
Brown et al. 2009 [63]	Exper OBS	10	Males with spinal cord injury (C3-T6); no diabetes	Yes	PPG - Indirect (BV) finger, hallux	LV Penis 100 Hz	To assess the reliability of using cutaneous blood flow and sweat release as markers of sympathetic activity during incipient autonomic dysreflexia induced by penile vibratory stimulation in men with spinal cord injury	Penile vibratory stimulation induced acute cutaneous vasoconstriction and minimal sweat release, accompanied by substantial elevations in systolic blood pressure and compensatory bradycardia in most quadriplegic and some paraplegic participants

Regarding measurement approach, 16 of the 23 studies reporting increased SBF (69.6%) used direct measurement techniques, and eight studies (34.8%) used surrogate measures (Table 3); these figures sum to more than 23 because one study, Clijsen et al. (2025), employed three distinct measurement methodologies, one direct and two indirect methods, and was therefore counted in both categories. Among the 20 studies that showed decreased SBF, four studies (20%) used direct measurement techniques whereas 16 (80%) used indirect methods (Table 3).

**Table 3:** Direction of SBF response-based measurement method SBF: Skin blood flow.

Measurement Type	Increased SBF	Decreased SBF	Mixed/No Significant Change
Direct	16*	4	2
Indirect	8*	16	1

Modality-based findings

SBF Measurement Methodologies

Measurement methodologies differ significantly across studies and can be grouped into direct and indirect measurements. Direct measurements of SBF explicitly quantify the blood perfusion or flow to the area of interest. Indirect measures of SBF infer perfusion based on physiologic outcomes like changes in skin temperature, volume, and muscle oxygenation. The direct measurements include laser Doppler flowmetry (LDF), laser speckle flowgraphy (LSFG), and laser Doppler imager (LDI). The indirect measures include dynamic infrared thermography (DIRT), infrared thermography (IRT), skin temperature (ST), skin temperature probe (STP), transcutaneous oxygen tension (TcPO2), near-infrared spectroscopy (NIRS), muscle oxygenation (SMO2), photoelectric plethysmography (PPG), and strain-gauge plethysmography (SGPG). Comparisons and interpretation across studies should be done with caution due to differences in SBF measurement methodologies.

Whole-Body Vibration

A significant increase in SBF was reported in most of the studies in healthy populations across varying ages [18-28]. Of the 12 studies examining the effects of passive WBV on SBF in healthy patients, 11 showed an increase in SBF after WBV. One study showed no statistical difference in SBF after WBV [29]. Certain studies examined WBV effects on SBF using indirect measures, such as proxy temperature, which has a direct correlation to the effect on SBF [25,26,28]. It is recognized that limitations may exist in translating the indirect measures of SBF to the degree of SBF changes caused by VT.

While most studies reported increases in SBF following WBV, two studies reported reductions, including one study utilizing whole body vibration exercise (WBVE), an active form of vibrational therapy, and another examining slow low-frequency motion exposure [30,31].

Local Vibration

Local vibration therapy (VT) refers to applying mechanical oscillations to the body at a specific frequency to specific anatomic sites using mechanical handheld percussion devices, such as massage guns, vibrating rollers, or motor-driven oscillatory systems. These devices deliver localized stimulation at varying frequencies and amplitude and target specific tissues rather than producing systemic effects.

Anatomical stratification

Lower Extremity

Local vibration applied to the lower extremity, such as the foot, gastrocnemius, and quadriceps, generally increased SBF. Studies delivering vibration to the first metatarsal head showed greater SBF responses at higher frequencies compared to lower frequencies or sham conditions [32-35]. Similar increases were observed when vibration was applied to different muscle groups and proximal regions, such as the gastrocnemius and vastus medialis, as well as the posterior thigh, with outcomes reported as increased skin temperature or perfusion-related measures [36,37].

However, some findings were inconsistent. One study reported no significant difference between vibrating and non-vibrating rollers, although increased blood flow oscillations were observed [38]. Another demonstrated reduced post-intervention SBF [39]. Additionally, one study reported that vibration applied under conditions of occlusive compression significantly reduced reactive hyperemia in the foot following pressure release [40].

Upper Extremity and Finger Blood Flow 

In contrast to lower extremity findings, vibration applied to the upper extremity, namely the hand and fingers, resulted in reductions in blood flow. Fifteen studies focused specifically on finger blood flow (FBF) and all demonstrated a significant reduction in finger blood flow following vibration exposure [41-55]. However, one study reported an increase in SBF on the dorsal surface of the hand following vibration exposure, whereas SBF on the palmar surface decreased [53]. Some studies also reported contralateral reductions in FBF when the ipsilateral hand was exposed to vibration, as well as further decreases associated with increased grip force during vibration exposure [42,43,46,48,52].

Influence of vibration parameters

SBF responses were influenced by vibration parameters, including frequency, magnitude, and exposure pattern. Higher frequencies were associated with increased SBF in the lower extremities and greater reductions in FBF. Reductions in FBF were influenced by vibration parameters, with higher vibration frequency and magnitude associated with a greater decrease in blood flow [41,44,45,47,49,50,54].

Vibration patterns also influenced outcomes. Intermittent vibration was associated with a significant increase in SBF in both diabetic and healthy subjects, whereas continuous vibration improved SBF only in healthy subjects [35]. However, one study with healthy participants found no significant difference in blood flow between hands exposed to continuous and intermittent vibration [51].

Clinical population-specific findings

Diabetes 

SBF responses were less pronounced in individuals with diabetes compared to healthy individuals [35,56]. In individuals with diabetes and suffering from neuropathy, SBF was reported to be increased following WBV [56-58]. Conversely, the individuals with Type II diabetes, along with the healthy/control participants, in this study showed decreased peripheral skin temperature and LE blood flow [59]. Additionally, as described above, an intermittent vibration pattern was associated with a significant increase in SBF, whereas continuous vibration did not show an improvement in SBF [35].

Restless Leg Syndrome (RLS)

The use of vibration therapy improved symptoms of RLS without a corresponding increase in SBF [60]. In contrast, a different study involving individuals with RLS reported a greater relative increase in SBF following WBV compared with age-matched healthy individuals, despite significantly lower baseline peripheral SBF [61].

Elderly 

In frail elderly individuals with multiple comorbidities, there were significant increases in skin surface temperature after low-frequency sound wave therapy, with the skin temperature serving as a proxy for changes in microvascular SBF [62].

Spinal Cord Injury

The use of local vibration on the penis in spinal cord-injured men resulted in a decrease in SBF [63]. 

Discussion

Summary of Key Findings

This review examined the physiological effects of external mechanical vibration on SBF in healthy participants and those with comorbidities. Across the literature, vibration is shown to be highly context-dependent and produce markedly different vascular responses depending on anatomical location (Table 4), vibration method, and patient pathology (Table 5). WBV and localized LE vibration generally increased SBF, whereas vibration to the hands and fingers predominantly reduced FBF. These opposing findings likely reflect the differences in vascular physiology, with vasodilatory endothelial and myogenic mechanisms predominating in the lower extremities and sympathetic mechanoreceptor-mediated vasoconstriction dominating in the hands and digits. Additionally, the response in clinical populations varied, with increased SBF observed in individuals with RLS and a more variable response seen in those with diabetes, reflecting the effects of disease on vascular tone and response. Collectively, these results suggest that mechanical vibration can alter cutaneous blood perfusion, with the magnitude and direction of the perfusion response varying by location, methodology and comorbidity.

**Table 4:** Direction of SBF response by site of vibration application Skin blood flow (SBF), whole-body vibration (WBV), "Predominant Exposure Model" indicates whether studies at each anatomical site were designed to model therapeutic/experimental vibration application or occupational vibration exposure (e.g., hand-transmitted tool vibration, grip force). The Digits/Hand category includes both exposure models; studies within this category are further differentiated in the Discussion (Upper Extremity and Occupational Considerations sections).

Anatomical Site	Increased SBF	Decreased SBF	Mixed / No Significant Change	Overall Pattern	Predominant Exposure Model
Digits/Hand	0	14	1*	Predominantly decreased	Mixed (Occupational + therapeutic/experimental)
Forearm	2	0	0	Predominantly increased	Therapeutic/experimental
Thigh/Buttock	2	0	0	Increased	Therapeutic/experimental
Penis	0	1	0	Decreased	Therapeutic/experimental
Lower Leg (Foot / Calf)	18	4	2	Predominantly Increased	Therapeutic/experimental
Unspecified WBV	1	1	0	Inconclusive	Therapeutic/experimental

**Table 5:** Direction of SBF response-based patient population/pathology Skin blood flow (SBF).

Patient Population	Increased SBF	Decreased SBF	Mixed/No Significant Change
Healthy	16	18*	3
Diabetes	4	1	0
Restless Leg Syndrome	2	0	0
Spinal Cord Injury	0	1	0
Frail Older Adults	1	0	0
Occupational Vibration Disease	0	1*	0

Most studies included in this review assessed acute, single-session changes in SBF rather than sustained or clinically validated therapeutic outcomes; an acute increase in SBF does not necessarily indicate a durable physiological adaptation or a meaningful clinical benefit, and where both were measured (e.g., in RLS), acute perfusion changes and symptom outcomes did not always track together. Additionally, a substantial proportion of reviewed studies, particularly those involving the digits, palmar hand, penis, and spinal cord injury populations, demonstrated vasoconstrictive or otherwise adverse vascular responses. These findings are not simply site-specific exceptions to an overall pattern of benefit; they represent a distinct and clinically important category of response that warrants equal consideration alongside vasodilatory findings.

The divergent SBF response between the lower extremities and the hands is unlikely to be explained by physiological mechanism alone. The measurement approach also differed systematically by site: lower-extremity studies more frequently used direct techniques (e.g., laser Doppler flowmetry) capable of detecting rapid local vasodilation, whereas upper-extremity and occupational studies relied heavily on strain-gauge plethysmography and other indirect surrogate measures sensitive to systemic sympathetic tone. This methodological divergence may compound, rather than confound, the observed anatomical divergence, as the techniques used at each site are differentially suited to detect the dominant mechanism proposed for that location.

WBV and localized vibration were associated with increased SBF in the lower extremities, while localized vibration applied to the palmar hand and fingers was associated with decreased SBF and a bilateral vasoconstrictive response, one study reported an opposing increase on the dorsal surface. Vasoconstrictive responses were also observed in the penis and, in individuals with spinal cord injury, in both the hands and feet. Response in clinical populations varied further: increased SBF was observed in individuals with restless leg syndrome, while diabetes produced more variable and often attenuated responses in either direction.

Modality-based interpretation

Whole Body Vibration

The literature indicates that WBV is associated with increased SBF in the lower extremities under most tested conditions; however, this response is frequency-dependent and not universal, as several studies report the opposite effect under different exposure conditions, which is discussed below. This trend is consistently observed across healthy populations of different ages across a range of frequencies. An increase is also observed in populations' diabetes and diabetic neuropathy, although the response magnitude differed between the two populations.

It was also found that individuals with RLS exhibited lower baseline SBF but experienced a greater increase in SBF following vibration therapy compared to healthy controls [61]. These findings suggest that while WBV generally increases SBF, underlying vascular dysfunction may influence the magnitude of the response. This is notable because diabetes-related endothelial dysfunction and reduced nitric oxide bioavailability would typically be expected to blunt vasodilatory capacity; the observed acute increase suggests vibration may transiently engage compensatory vasodilatory pathways in this population, though whether this translates into sustained improvement in vascular function has not been established.

Additionally, in a unique approach, a study investigating the effects of sound wave therapy (sinusoidal sound waves 27-113 Hz), a gentler vibratory approach for the elderly population, reported increases in skin temperature, suggesting improvements in blood circulation [62]. These findings suggest that modified, lower intensity forms of vibration therapy may be associated with circulatory changes in populations unable to tolerate traditional WBV interventions, though this was assessed in a single study and should not be interpreted as establishing comparative efficacy between modalities.

The mechanisms behind these increases are attributed to the activation of the myogenic response of the vessels [21]. While this response is not exclusive to WBV and can occur with various forms of mechanical or muscular stimulation, WBV induces rapid, small-scale muscle contractions that promote vasodilation and increase the delivery of oxygenated blood to distal extremities [26]. In contrast, very slow, low-frequency WBV (0.03-0.2Hz) and WBVE, where exercise was used to produce WBV have been associated with peripheral vasoconstriction and decreased SBF [30,31]. It is suggested that the vasoconstriction seen with exposure to low-frequency motion is due to a change in sympathetic outflow [31]. Similarly, the decrease in SBF seen with exercise was suggested to be due to blood shunting to more vital organs, a sympathetic response.

Although most studies reported an increase in SBF, these variations in findings suggest a frequency-dependent vascular response to WBV, potentially mediated by an interaction between myogenic vascular mechanisms and sympathetic nervous system activity.

Anatomical differences in SBF response

Lower Extremity (Local VT)

The literature indicates that local vibration applied directly to the lower extremities increases SBF to the treated areas.

A notable difference in the literature concerns vibration patterns. It was discovered that intermittent vibration followed by short pauses significantly increased SBF in both healthy and diabetic subjects [35]. Continuous vibration only improved flow in healthy participants. This suggests that the microvasculature in diseased states may require a brief period of recovery to respond to the stimulus. This finding highlights the importance of intervention design. 

The magnitude of SBF response to vibration appears to be influenced by vibration parameters, particularly frequency. Higher frequencies have been associated with larger increases in skin perfusion compared to lower frequencies [33]. However, physiological context also plays an important role. Although weight-bearing activity is typically associated with reduced plantar circulation, one study demonstrated that the application of vibration applied during weight-bearing activity improved SBF while standing [32]. In contrast, other studies demonstrated reductions in post-intervention SBF and attenuated reactive hyperemia, suggesting that vascular response may vary depending on mechanical stimulus and baseline physiological state [39,40]. Thus, vibration parameters and loading conditions may significantly influence SBF in the lower extremities.

Upper Extremity (Local VT)

As noted in the Introduction, upper extremity vibration studies fall into two categories with distinct intent and exposure design: controlled experimental studies of vibration as a therapeutic or diagnostic modality, and studies modeling occupational hand-transmitted vibration exposure. Although both consistently report reduced FBF in the palmar hands and fingers, they are considered separately where relevant below and in the Occupational Considerations section.

In contrast to the increase in blood perfusion observed in the lower extremities following WBV or local vibration, the upper extremities, specifically the palmar fingers, consistently showed reduced blood perfusion in response to local vibration. Multiple studies have demonstrated a dose-dependent response, in which higher vibration frequency, magnitude, or duration resulted in greater reductions in FBF [44,46,48-50,54]. Additionally, increasing the contact surface area of the vibration probe further amplified the reduction in FBF [43]. Women, who have lower vibrotactile thresholds than their male counterparts, showed greater reductions in FBF [42], and increasing the contact surface area of the vibration probe amplified these reductions [43].

This response likely reflects a sensory-mediated vasoconstrictive mechanism involving the activation of mechanoreceptors, such as Pacinian corpuscles, which are more densely distributed on the palmar surface of the hand [41]. Given the increased density of mechanoreceptors on the palmar surface, the pronounced decrease in FBF likely reflects an enhanced mechanoreceptor activation and a resultant sympathetic-mediated vasoconstriction. 

Regional differences were observed when vibration was localized to the hand while gripping a vibratory handle, which then resulted in increased SBF to the dorsal aspect, while decreasing SBF to the palmar phalanges [53]. In contrast to the palmar surface, the dorsal surface of the hand contains fewer mechanoreceptors and a different vasculature composition, which may account for the increased, or divergent, SBF response. In a related finding, localized forearm vibration increased SBF in both diabetic and healthy individuals [56], further reinforcing the role of regional anatomical differences.

This apparent discrepancy in the upper extremities may again be explained by regional differences in mechanoreceptor density and vascular anatomy between the forearm and the digits, suggesting that vibration-induced cutaneous perfusion is highly site-specific and dependent on mechanoreceptor density and activity. These findings indicate that vibration-induced cutaneous perfusion is highly site-specific and dependent on mechanoreceptor density and activity.

Interestingly, ipsilateral vibration can produce a contralateral FBF response [41,43,46,48]. For example, a 125 Hz vibration applied to the right hand reduced the FBF in both hands. These findings support a role for central sympathetic mechanisms in the vascular response to vibration [43,64].

Influence of vibration parameters

SBF responses were influenced by vibration parameters, including frequency, magnitude, and exposure pattern. Higher vibration frequencies were associated with increased SBF in the lower extremities, but greater reductions in finger blood flow (FBF). Increased vibration magnitude was similarly associated with greater decreases in FBF. Vibration pattern also influenced outcomes, with intermittent vibration increasing SBF in both healthy and diabetic individuals, whereas continuous vibration improved SBF primarily in healthy populations. However, some studies reported no significant difference between continuous and intermittent exposure. Trends suggested that intermittent exposure might yield slightly less pronounced reductions, consistent with previous studies showing partial recovery of blood flow during breaks from exposure [52]. However, one study reported no significant difference between continuous and intermittent exposure’s effect on blood flow between hands, suggesting that the effect of vibration pattern may depend on study design and experimental conditions [51].

Mechanistic interpretation

The physiological mechanisms that mediate the effects of vibration on SBF appear to be multifaceted and dependent on both the treatment modality and anatomical site involved.

In WBV, increases in SBF are primarily attributed to activation of the myogenic response [21] and repeated muscle contractions [26]. These mechanical stimuli facilitate vasodilation and enhance peripheral circulation [18,27]. Notably, observed increases in SBF with WBV in individuals with diabetes have been shown to occur through a nitric oxide (NO)-independent pathway, which suggests that alternative vasodilatory mechanisms may exist under these circumstances [59].

In contrast, LV applied to the lower extremities appears to increase SBF through distinct mechanisms. LV- induced increases in SBF may be endothelial and NO-dependent [58], with shear stress-mediated mechanisms contributing to improvements in microvascular circulation. Zhu et al. [32] demonstrated that LV-mediated increases in SBF in the LE are regulated through both endothelial and neurogenic mechanisms. Additionally, preconditioning with vibration has been shown to alter the normalization of SBF following walking exercise via metabolic and neurogenic controls without producing significant changes in the myogenic, respiratory, or cardiac frequency bands [39].

The vasomotor response to vibration in the upper extremities, particularly the digits, appears to differ fundamentally from that observed in the lower limbs. Exposure to vibration at this site is associated with a reduction in FBF, potentially due to a sensory-mediated vasoconstriction. This response has been attributed to the activation of the mechanoreceptors, particularly Pacinian corpuscles, which are thought to elicit a sympathetic response and therefore vasoconstriction [31,63,64]. This mechanism is further supported by observations of bilateral and contralateral reductions in FBF following unilateral vibration exposure, which implicates the involvement of central sympathetic pathways rather than a pure local vascular response [41-43]. The degree of vasoconstriction has been shown to correlate with vibrotactile perception thresholds, and as women demonstrate lower vibrotactile perception thresholds than men, they correspondingly experience a greater reduction in FBF during vibration exposure [42].

It is also worth distinguishing the study designs underlying these mechanistic claims: sympathetic vasoconstriction in the upper extremities has been inferred from both therapeutic/experimental studies and occupational-exposure models, which may not be mechanistically identical despite producing similar directional changes in FBF. Because most cited mechanisms are derived from acute, single-session exposures, it also remains unclear whether these pathways reflect transient reflexive responses or would persist with repeated or chronic exposure. Collectively, these findings indicate that the physiological response to vibration is highly site-specific and dependent on the underlying neural and vascular mechanisms involved. It should be noted, however, that these mechanisms are proposed based on observed hemodynamic patterns, as direct mechanistic measurements were limited across the reviewed literature.

Clinical population-specific findings 

SBF responses to vibration differed across clinical populations, suggesting that underlying pathology influences the magnitude and direction of the response (Table 5).

Diabetes

The effect of vibration on SBF in individuals with diabetes was inconsistent, with studies reporting both increased and decreased perfusion depending on vibration parameters, anatomic location, and measurement method. For example, one WBV protocol (26 Hz, 2mm, 300 s) measured with direct techniques increased foot SBF in individuals with diabetic sensorimotor polyneuropathy, whereas another protocol (24 Hz, 4 mm, 360 s) assessed using indirect techniques reported reduced plantar perfusion in individuals with type 2 diabetes [57,59]. Despite only modest changes in the protocol, differences in vibration characteristics, participant populations and methods of assessing perfusion may all have contributed to the contrasting findings. These results suggest that vascular response to vibration in individuals with diabetes is heterogeneous and may be influenced by both treatment parameters and methodological differences, highlighting the need for a standardized protocol and outcome measures. Individuals with diabetes mellitus face microvascular dysfunction from hyperglycemia-induced endothelial damage and oxidative stress that can lead to peripheral neuropathy and affect vascular function. As a result, vascular responses to vibration appear variable and often attenuated compared to healthy individuals, likely reflecting impaired microvascular function.

Restless Leg Syndrome

The available evidence suggests that WBV acutely enhances SBF in individuals with RLS but differ in their findings regarding baseline perfusion and long-term changes. One study found that participants with RLS had higher baseline SBF than healthy controls and exhibited a greater immediate increase in SBF following WBV, despite NO concentrations remaining unchanged [61]. This suggests that mechanisms other than NO release may mediate the vasodilatory response. Conversely, the follow-up study measured the RLS participant SBF in the evening, which is when RLS symptoms are most severe, and found that the baseline SBF was decreased compared to healthy controls [56]. In this study, WBV was shown to produce acute increases in SBF, but repeated treatment over a two-week intervention did not increase resting SBF despite symptom improvements. One proposed mechanism underlying RLS is an urge to move the legs to relieve tissue hypoxia from impaired venous structure and vibration has been shown to improve oxygenation and reduce symptoms [65]. Together, this suggests that WBV may improve oxygen delivery to peripheral tissues, thereby minimizing discomfort associated with RLS a, however, sustained symptom improvement does not appear to be dependent on consistent elevations in resting perfusion.

Elderly 

Sound wave vibration was associated with increases in skin temperature, which was used as a proxy measure for SBF [62]. This vibration therapy showed improvements in mobility among the elderly, which may have been related to the enhanced LE blood flow. However, the authors also suggest this increased mobility may also be due to increased social interaction during the study rather than the vibration.

Spinal Cord Injury 

Individuals with spinal cord injury exposed to localized penile vibration resulted in decreased SBF in the hands of participants with quadriplegia and feet of both quadriplegic and paraplegic participants [63]. This reduction reflected a sympathetically mediated vasoconstrictive response associated with autonomic dysreflexia, which occurred alongside a marked increase in blood pressure and decreased heart rate. Like other disease states, this finding highlights that the vascular response is highly dependent on the underlying pathology, including neurological status. However, given the predominant increase in SBF LE response outlined above, these findings suggest that disrupted autonomic control following a spinal cord injury can override the local vasodilatory effect of vibration observed in healthy populations, particularly in the lower extremities.

Occupational considerations: vibration and grip effects 

This section addresses studies specifically designed to model real-world occupational vibration exposure as distinct from the experimental/therapeutic vibration studies discussed above. These occupational-exposure studies consistently demonstrated reductions in FBF during localized hand vibration [45,49,52,55]. However, the findings regarding the influence of grip force and static load meant to replicate occupation exposure remain inconsistent. Some studies report no significant changes in FBF when only a passive static load was applied without vibration, whereas other studies showed that a larger grip force further decreased FBF even in the absence of vibration [49,55]. Methodological differences may explain the discrepancy. For example, a 10 N grip force reduces FBF, likely due to mechanically impeded blood flow, whereas a passive 10 N static load produces no significant effect on FBF [49,55]. Such differences may influence how accurately laboratory findings reflect real-world occupational exposure.

Studies designed to better replicate occupational settings that utilize continuous equipment-induced hand vibration further demonstrated that higher grip forces combined with vibration lead to greater reductions in hand blood flow [52,55].

The decrease in FBF with finger vibration is consistent with the suggested pathophysiology of vibration white finger (VWF). VWF occurs when repeated occupational vibration exposure causes excessive vasoconstriction and leads to blanching of the finger, accompanied by numbness and pain [64]. The underlying pathogenesis is believed to involve both central sympathetic hyperreactivity and local vascular changes mediated by vasoactive factors [64]. Mechanoreceptors in the palmar hand, such as Pacinian corpuscles, may mediate this response through vibration-induced sympathetic vasoconstriction.

Vibration effects on reactive hyperemia

While most studies report increased blood flow, some suggest that vibration can mitigate the reactive hyperemic response. Vibration, particularly when combined with compression, reduces SBF and the hyperemic response [39,40]. It is suggested that this vibration-induced vasoconstriction may be positively correlated with a simultaneous increase in blood pressure [31,63]. This contrasts with vasodilation observed in WBV studies but is consistent with vasoconstriction seen in finger vibration. Short-term vibration may decrease peak hyperemia, while prolonged exposure may reduce digital blood flow [48].

Study limitations

Review Process Limitations

The findings of this scoping review must be considered taking into account some methodological limitations. Although three of the most prominent databases (Ovid, Embase, Web of Science) were used, omitting engineering databases such as IEEE Xplore may have limited the identification of relevant papers. Similarly, additional databases including Scopus, CINAHL (Cumulative Index to Nursing and Allied Health Literature), SPORTDiscus, and the Cochrane library were not searched. As with the omission of engineering databases, we believe most relevant studies addressing SBF as a physiological outcome would be captured within the three databases searched; however, we acknowledge that some studies within rehabilitation, sports medicine, and physiotherapy literature may be underrepresented as a result. This configuration represents a high degree of systematic evidence synthesis, combining Ovid and Embase’s comprehensive coverage of clinical and physiologic literature while utilizing Web of Science’s interdisciplinary citation network to minimize the risk of unintentional exclusion of relevant studies. Confining the search to English-language publications may also miss some relevant data. Despite efforts to be comprehensive, the omission of unpublished trials, abstract-only papers, and other gray literature may skew results towards positive outcomes. The review process was also constrained by grouping vibration delivery methods into whole-body vibration and local vibration therapy, as this generalization may obscure the identification of the most effective form of therapy.

Additionally, the nature of a scoping review causes data synthesis to be reliant on proportion-based summaries rather than pooled effect estimates. This hinders the ability to draw definitive conclusions regarding overall treatment efficacy. These proportion-based findings (e.g., the percentage of studies reported increased versus decreased SBF) aggregate results across studies with different vibration parameters, measurement techniques, anatomical sites, and participant populations, and should therefore not be interpreted as weighted or pooled effect estimates of vibration's impact on SBF. Similarly, because vibration frequency and exposure duration varied too widely across studies and modalities to permit meaningful tabular stratification, these variables are addressed narratively rather than through structured subgroup summaries; this represents a limitation relative to the anatomical, population-based, and measurement-based stratifications provided elsewhere in this review.

Limitations of the Included Studies

The evidence constituting this review is constrained by several factors present throughout the included studies. Primarily, there is a lack of generalization in vibration protocols. There appears to be no generalized “dosage” of therapy. This includes variations in frequency, delivery method (WBV vs. local), application pattern (continuous vs. intermittent), and measurement methods (direct vs. indirect). Furthermore, most studies had small sample sizes, with 27 involving 15 or fewer participants. There are also researchers and subject-blinding challenges with VT, as the mechanical nature of the therapy makes true blinding impossible when compared with no vibration, since sham treatments are not possible. Therefore, most studies in this review may be subject to performance bias. As mentioned above, limitations also exist with the use of surrogate markers as a marker for SBF when direct SBF measurements were unavailable [25,28,29]. This would most likely have the greatest effect when investigating the degree of change in SBF caused by VT. Lastly, geographic clustering is evident, with most studies originating from Europe or the United States, though this is likely due to methodological limitations, as discussed above.

Gaps in the literature and future research directions

There appears to be a promising link between vibration therapy and increased blood flow. However, several gaps exist. There is a lack of consensus on the therapeutic details of vibration, specifically variables such as frequency, acceleration, and amplitude. Higher magnitudes may impede blood flow [44,47-49,54,55]. Future studies are needed to establish potential clinical guidelines and determine the therapeutic window for the treatment parameters.

Second, while studies have yielded valuable data on short-term hemodynamic changes in patients using vibration, future studies should determine whether these acute changes in SBF will translate into clinically relevant outcomes. Addressing this disconnect is a key research opportunity, as currently, the link between laboratory-observed SBF changes and therapeutic efficacy is speculative.

Third, underlying biological mechanisms should be explored in greater detail. This review found early evidence that suggested vibration modulates nitric oxide levels [56,57,61], but specific pathways remain vague. Further, it was found that the central sympathetic pathway [43], and Pacinian corpuscles [41] may contribute to the microcirculatory pathway, but specifics about these links remain vague.

Implications for practice, policy, and research

The findings of this review highlight VT as a priority area for rigorous investigative research. While the potential for VT as a non-invasive clinical intervention exists, current evidence primarily consists of speculative interpretations of laboratory experiments. Therefore, research should prioritize longitudinal studies over clinical implementation at this stage. Research in this area is essential to establish whether the observed physiological responses can be used as a reliable therapeutic intervention in patients with local ischemia or small-vessel disease.

## Conclusions

The findings of this scoping review characterize vibration therapy as a physiological stimulus with a measurable acute effect on SBF that is influenced by delivery method, anatomical site, and specific treatment parameters. While the current literature suggests potential for VT to modulate microcirculation, these observations are based primarily on transient, single-session laboratory data rather than sustained or clinically validated outcomes. Therefore, VT should be viewed as a high-priority area for future research rather than an established clinical intervention. To move beyond speculative interpretation of data, future studies must investigate whether acute, laboratory-observed changes in SBF translate into meaningful outcomes for tissue viability and wound healing. Establishing this link is a prerequisite for defining the therapeutic potential of VT in a clinical setting.
